# Explainable artificial intelligence for botnet detection in internet of things

**DOI:** 10.1038/s41598-025-90420-6

**Published:** 2025-03-04

**Authors:** Mohamed Saied, Shawkat Guirguis

**Affiliations:** Institute of Graduate Studies & Research, Alexandria University, 832, Elhorrya Road, Alexandria, 21526 Egypt

**Keywords:** Internet of things, Machine learning, Cyber security, Botnet detection, Explainable artificial intelligence, Computational biology and bioinformatics, Computational models, Machine learning, Network topology

## Abstract

The proliferation of internet of things (IoT) devices has led to unprecedented connectivity and convenience. However, this increased interconnectivity has also introduced significant security challenges, particularly concerning the detection and mitigation of botnet attacks. Detecting botnet activities in IoT environments is challenging due to the diverse nature of IoT devices and the large-scale data generated. Artificial intelligence and machine learning based approaches showed great potential in IoT botnet detection. However, as these approaches continue to advance and become more complex, new questions are opened about how decisions are made using such technologies. Integrating an explainability layer into these models can increase trustworthy and transparency. This paper proposes the utilization of explainable artificial intelligence (XAI) techniques for improving the interpretability and transparency of the botnet detection process. It analyzes the impact of incorporating XAI in the botnet detection process, including enhanced model interpretability, trustworthiness, and potential for early detection of emerging botnet attack patterns. Three different XAI based techniques are presented i.e. rule extraction and distillation, local interpretable model-agnostic explanations (LIME), Shapley additive explanations (SHAP). The experimental results demonstrate the effectiveness of the proposed approach, providing valuable insights into the inner workings of the detection model and facilitating the development of robust defense mechanisms against IoT botnet attacks. The findings of this study contribute to the growing body of research on XAI in cybersecurity and offer practical guidance for securing IoT ecosystems against botnet threats.

## Introduction

IoT has revolutionized various industries, bringing unprecedented levels of connectivity and convenience. However, this ubiquitous interconnectivity has also exposed IoT ecosystems to a myriad of security risks, with botnet attacks being a significant concern. Botnets, consisting of networks of compromised devices controlled by malicious actors, pose a severe threat to the integrity and privacy of IoT deployments. Detecting and mitigating botnet activities in real-time is essential to safeguard IoT systems and protect sensitive information.

Constrained resources of IoT devices compromise its capabilities when installing traditional security software. An increasingly growing research focuses on involving “intelligence” into the security systems and leveraging artificial intelligence (AI) techniques to classify and detect malicious traffic. AI-driven intrusion detection systems can autonomously analyze vast amounts of network traffic, identify malicious patterns, and adapt to evolving attack vectors. Machine Learning (ML), a subset of AI, has emerged as a powerful approach for IoT intrusion detection^1^. ML algorithms can learn from historical data, extract meaningful features, and build models that can differentiate between normal and malicious network behavior. While AI offers tremendous potential for IoT intrusion detection, several challenges need to be addressed. The scarcity of labeled training data, the need for real-time processing and low latency, and the interpretability of complex AI models are some of the key challenges to overcome. Additionally, the dynamic nature of IoT environments requires continuous adaptation of AI models to new attack vectors and evolving network topologies.

In our previous work^2^, we comparatively analyzed six tree-based ML algorithms i.e. decision trees (DT), Random Forest (RF), bagging meta classifier (BMC), adaptation boosting (ADB), gradient descent boosting (GDB), and extreme gradient boosting (XGB). The study provided an empirical assessment of the effectiveness of tree-based methods in identifying intruders within IoT networks. It examined the performance of both bagging and boosting techniques for detecting botnets and carried out a comprehensive experimental benchmark to evaluate their effectiveness. We addressed the challenge of botnet detection in IoT environments. RF model demonstrated the best performance in accurate binary classification of network traffic into benign and malicious. However, the interpretability of ML models remains a crucial aspect that needs to be addressed to gain trust and understanding from stakeholders, such as network administrators, security analysts, and end-users. Understanding the factors contributing to the detection of botnet activities is crucial to take appropriate remedial actions and devise effective countermeasures. As the complexity of mathematical decisions made by these models increases, it becomes increasingly challenging to articulate the underlying reasoning. Moreover, this mathematical abstraction fails to instill confidence in users regarding the decision-making process of a specific model.

XAI has emerged as a field of research that focuses on developing methods and techniques to elucidate the decision-making process of complex ML models. XAI aims to bridge the gap between the opacity of black-box models and the need for transparency and interpretability in critical domains. In this paper, we extend our previous research on botnet detection in IoT^2^ by integrating XAI techniques into the RF model. Our objective is to enhance the interpretability and transparency of the botnet detection process, enabling stakeholders to comprehend the rationale behind the model’s decisions. The integration of XAI in the botnet detection process offers several advantages. Firstly, it enhances the interpretability of the RF model, enabling network administrators and security analysts to gain a deeper understanding of how the model identifies botnet activities. This understanding can facilitate the development of more effective defensive strategies to mitigate the impact of botnet attacks. Secondly, XAI explanations provide transparency and trust, allowing stakeholders to verify the fairness and reliability of the detection system. Moreover, the interpretability provided by XAI techniques can aid in the early detection of emerging botnet attack patterns, empowering organizations to proactively adapt their security measures. To the best of author’s knowledge, the proposed study is the first study to have used XAI to detect botnet attacks on IoT environments. This paper presents the experimental results that demonstrate the effectiveness of the proposed approach. It evaluates the interpretability of the RF model through the generated explanations and assesses the impact of incorporating XAI in the botnet detection process. Moreover, it leverages these interpretability features to gain insights into the factors contributing to botnet activities in IoT networks.

The primary contributions of this paper can be summarized as follows:

1. Examining the literature of using trees based algorithms in binary classification of IoT network intrusion.
2. Employing the XAI techniques within the botnet detection framework to enhance interpretability. By employing feature importance analysis and visualization techniques, the proposed approach provides insights into the critical features driving the botnet detection predictions, enabling security analysts to gain a deeper understanding of the underlying botnet behaviors.
3. Proposing a novel XAI-based botnet detection framework for IoT networks.
4. Conducting an extensive experimental evaluations on a real-world IoT dataset to assess the effectiveness of the proposed XAI-based botnet detection framework.

The structure of the remaining sections in this paper is as follows: “Motivation” section highlights the motives behind conducting this research. “Related work” section provides a comprehensive review of existing literature on botnet detection in IoT networks, highlighting the contributions and limitations of previous approaches. In “Material and methods” section, we present the materials and methods employed in this paper. “Experiments and results” section presents the experimental results of three employing XAI different techniques. Finally, “Conclusion and future work” section concludes the paper, summarizing the contributions and findings of this study.

## Motivation

The increasing adoption of IoT devices has revolutionized various domains, ranging from smart homes and healthcare to industrial automation and transportation. However, the rapid growth of IoT networks has also introduced significant security challenges, with botnet attacks emerging as a major threat. The motivation behind this research stems from the urgent need to detect and mitigate botnet activities in IoT environments to safeguard the privacy, security, and integrity of these interconnected systems.

Privacy and Security Risks: Botnet attacks pose severe risks to the privacy and security of IoT networks^3^. Compromised devices within a botnet can be exploited to collect sensitive information, such as personal data, financial credentials, or proprietary industrial data. Unauthorized access to IoT devices can lead to privacy breaches and misuse of personal or sensitive information. By detecting and preventing botnet activities, the privacy and security of IoT ecosystems can be safeguarded, ensuring data confidentiality and minimizing the potential for unauthorized access.

Network Performance and Availability: Botnet attacks often involve coordinated efforts to overload network resources, leading to degraded performance and disruptions in IoT services. Distributed denial-of-service (DDoS) attacks, for instance, can flood the network with an overwhelming volume of traffic, rendering IoT devices and services unavailable to legitimate users^3^. By effectively detecting and mitigating botnet activities, the overall network performance and availability of IoT services can be improved, ensuring uninterrupted operations and a seamless user experience.

IoT Device Integrity: Botnet attacks exploit vulnerabilities in IoT devices, compromising their integrity and potentially turning them into malicious actors within the network. Infected devices may exhibit abnormal behaviors, consume excessive resources, or engage in unauthorized communication, jeopardizing the reliability and trustworthiness of the entire IoT ecosystem. By accurately detecting botnet activities, compromised devices can be identified and isolated, preventing further propagation of the attack and preserving the integrity of IoT device networks.

Regulatory and Compliance Requirements: With the increasing awareness of cybersecurity risks in IoT deployments, regulatory bodies and industry standards are placing greater emphasis on ensuring the security and resilience of IoT networks. Compliance with these regulations necessitates the implementation of robust botnet detection mechanisms. By employing explainable machine learning models, such as Explainable XGB, organizations can not only meet the compliance requirements but also provide transparent and interpretable evidence of botnet detection to regulatory authorities.

Advancements in Explainable Artificial Intelligence (XAI): The need for interpretability in machine learning models has gained significant attention, especially in security-critical domains^4^. Traditional machine learning approaches often lack transparency, making it difficult to understand the reasoning behind their predictions. The emergence of XAI techniques offers an opportunity to bridge this gap by providing insights into the decision-making process of complex models, enabling security analysts to comprehend the factors contributing to botnet activities^5^. By leveraging the interpretability features, this research aims to enhance the trust and acceptance of machine learning models for botnet detection in IoT networks.

In conclusion, the motivation behind this research lies in addressing the pressing need for effective botnet detection in IoT networks. By mitigating the privacy and security risks, improving network performance and availability, preserving IoT device integrity, meeting regulatory requirements, and leveraging advancements in XAI, this research aims to contribute to the development of a comprehensive and interpretable botnet detection framework.

## Related work

For efficient AI based intrusion detection in IoT environment, a number of studies have been conducted. ML is a type of artificial intelligence, which uses several learning algorithms to train the data without the need for intensive programming algorithms. It has been used extensively for classification tasks. Tree based ML includes a broad family of techniques such as DT, RF, and ensemble learning (EL). They have been successfully applied to solve challenging problems in a number of fields^1^. This section presents the most relevant related work and discusses the related research studies.

A number of research works proposed adopting DT algorithm for network intrusion detection in IoT environment. Doshi et al.^6^ created a labeled training dataset by simulating a local network of consumer IoT devices, which included both benign and malicious traffic. The authors employed a labeled dataset to assess the performance of five distinct machine learning classifiers: KNN, LSVM (SVM with linear kernel), an ANN with a four-layer fully-connected feed-forward architecture, DT, and RF using Gini impurity scores. The researchers noted that incorporating stateful features resulted in improved accuracy compared to using stateless features alone. RF exhibited the highest accuracy of 99.8%, surpassing KNN and DT with accuracies of 99.5% and ANN with an accuracy of 98.9%.

On the other hand, LSVM had the worst accuracy of 92.1%. Anthi et al.^7^ proposed a supervised approach consists of three layers for detecting and classifying intrusions in IoT. The system is performing three main functions: formulate a profile for the normal behavior of each IoT device. It identifies malicious packets on the network in case of attack, then it classifies the type of the attack. They built a smart home test bed consisting of 8 IoT devices. 12 attacks were injected with four main categories i.e. DoS, man in the middle (MITM), reconnaissance, and replay. To classify unseen data, nine classifiers were selected. The selection of classifiers was based on the classification time, ability to support multi class classification, and high dimensional feature space. The nine classifiers are Naive Bayes, Bayesian Network, J48, Zero R, One R, Simple Logistic, SVM, Multi-Layer Perceptron (MLP), and Random Forest. The findings indicated that the DT J48 model achieved the highest performance level. The reported evaluations for the three functions device profiling, detecting wireless attacks and attack type classification are 96.2%, 90.0%, and 98.0% respectively.

Goyal et al.^8^ presented an approach for detecting botnets based on behavioral analysis. They evaluated Logistic Regression, SVM, ANN, and DT. The achieved accuracy rates were 99.23%, 99.86%, 99.74% respectively, without reported results for DT. Chaudhary and Gupta^9^ presented ML based framework for detecting DDoS attack working in two phases: Detection phase and Mitigation phase. They used dataset by capturing using Wireshark the traffic of IoT environment consisted of PCs and Raspberry Pi. It contains a total of 114,565 packets, 10,061 of them are benign. For classification, they evaluated four algorithms Random Forest, SVM, Logistic Regression, and DT. The reported accuracy rates are 99.17%, 98.06%, 97.50%, and 98.34% respectively.

Another research direction proposed adopting RF algorithm for network intrusion detection in IoT. Alsamiri and Alrashdi et al.^10^ proposed an NIDS for loT in a smart city based on RF algorithm and Extra Tree (ET). To evaluate their model, UNSW-NB15^11^ dataset was used. They reported a detection accuracy of 99.34% with lowest false positive rate.

Thamilarasu et al.^12^ demonstrated mobile agent based intrusion detection system to medical IoT. They simulated a hospital network topology of Internet of Medical things. They trained five supervised ML algorithms i.e. SVM, DT, Naïve Bayes, KNN, and Random Forests. They reported unsuitable performance for KNN and Naïve Bayes algorithms. While, SVM, DT, and RF performed well. The best reported performance was for RF as it achieved an approximate accuracy of 100%.

Hammoudeh and Aljaberi^13^ proposed intrusion detection system for IoT based on the gated recurrent unit (GRU) deep learning algorithm with flower pollination algorithm (FPA) for feature selection with an accuracy of 99.59%.

To evaluate their approach, an extensive experimental analysis was conducted, comparing it against several ML-based models such as DT, RF, LR, and an ensemble of SVM, DT, RF, LR, and GDB algorithms. The achieved accuracy rates were 91.04%, 89.39%, 90.37%, and 92.03%, respectively. The study utilized the KDD Cup 99 dataset^14^; however, it is worth noting that this dataset is outdated and does not specifically address IoT network intrusions, including HTTP DoS or botnet attacks.

Boosting techniques for network intrusion detection in IoT has been adopted by a few studies. Kumar et al.^15^ used a two-step process for identifying Peer to peer P2P bots which are detection step and analyzing step. For the classification step, tenfold cross validation is used on RF, DT and XGB. Their approach achieved detection rate of 99.88%. For P2P botnet detection, the model was trained using traffic data from three specific botnets: Waledac, Vinchuca, and Zeus. Hazman et al.^16^ proposed an approach for intrusion detection in IoT-based smart environments with Ensemble Learning called IDS-SIoEL. Their methodology incorporates ADB and combines various feature selection techniques, including Boruta, mutual information, and correlation. They evaluated their approach on IoT-23, BoT-IoT, and Edge-IIoT datasets. They reported a detection accuracy of 99.90%.

Khan et al.^17^ proposed a proactive interpretable prediction model using to detect different types of security attacks using the log data generated by heating, ventilation, and air conditioning (HVAC) attacks. Several ML algorithms were used, such as DT, RF, GDB, ADB, Light Gradient Boosting (LGB), XGB, and Categorical Boosting (CAB). According to their findings, the XGB classifier yielded the highest accuracy of 99.98%. The study utilized the Elnour et al. dataset.

In the field of IoT healthcare, Ashraf et al.^18^ proposed a federated intrusion detection system. Their approach relies on employing lightweight artificial neural networks in a federated learning manner. In addition, it uses Blockchain technology to provide a distributed ledger for aggregating the local weights and then broadcasting the up-dated global weights after averaging. They conducted a comparison between ANN and XGB models using the BoT-IoT dataset. The results show that ANN has better performance of 99.99 rather than XGB of 98.96.

Alissa et al.^19^ proposed a decision tree, an XGB model, and a logistic regression (LR) model. They used UNSW-NB15 dataset with applying features correlation technique resulting in discarding nine features. They reported that the decision tree outperformed with 94% test accuracy with slight higher accuracy that XGB while LR achieved the worst accuracy.

Garg et al.^20^ conducted a performance comparison between boosting techniques and non-boosting ensemble-based techniques. They categorized two types of attacks: IoT attacks and DDoS attacks, as binary class and multiclass outputs, respectively. The evaluation was carried out using three datasets: BoT-IoT, IoT-23, and CIC-DDoS-2019. Two boosting methods, XGB and LGB, were employed. Among them, LGB achieved the highest performance, with an accuracy of 94.79%.

Bhoi et al.^21^ proposed a model based on LGB for detecting anomalies in an IoT environment. For optimizing the hyper parameters of LGBm they adopted the Gravitational Search-based optimization (GSO). They compared the optimization results with Particle Swarm Optimization (PSO). The evaluation was conducted using a simulated IoT sensors dataset called the IoT dataset. They reported an optimal accuracy of 100%.

Few studies in the literature adopted boosting techniques for botnet detection in IoT. Saied et al.^22^ presented a comparative analysis for five boosting based algorithms in binary-class classification i.e. ADB, GDB, XGB, CAB, Hist Gradient Boosting (HGB). Their analysis showed that HGB outperformed with 99.99% of detection accuracy on N-BaIoT dataset with 115 features. They presented another study^23^ evaluating six boosting based algorithms in multi-class classification i.e. ADB, GDB, XGB, CAB, HGB, and LGB. The HGB achieved the highest detection accuracy on N-BaIoT dataset with 115 features.

In summary, the studies to date have utilized several datasets and algorithms to detect intrusions in IoT. However, to the best of author’s knowledge, no previous studies have used XAI to detect botnet attacks on IoT environments. A comparative analysis of the previous related work is presented in tabular form in Table 1.

**Table 1:** Comparative analysis for the related work.

Algorithm	Author	Ref	Year	Dataset	Objective	No of classes	No of features	Accuracy
RF	Doshi	^6^	2017	Simulated	DDoS detection in IoT	2	11	99.80
DT (J48)	Anthi	^7^	2019	Simulated	Intrusion detection in smart medical IoT	2	121	99.00
DT	Goyal	^8^	2019	Simulated	Detecting botnets based on behavioral analysis in IoT	2	3	87.15
DT	Chaudhary	^9^	2019	Simulated	DDoS detection in IoT	2	NA	98.34
RF	Chaudhary	^9^	2019	Simulated	DDoS detection in IoT	2	NA	99.17
RF + ET	Alrashdi	^10^	2019	UNSW-NB15	NIDS for IoT	2	49	99.34
RF	Thamilarasu	^12^	2020	Simulated	Intrusion detection for medical IoT	2	NA	100.0
RF	Hammoudeh	^13^	2021	KDDCup99	NIDS for IoT	2	41	89.39
XGB	Kumar	^15^	2019	Synthetic	Peer-to-Peer Botnet Detection	2	18	99.88
EL ADB	Hazman	^16^	2022	IoT-23, BoT-IoT, Edge-IIoT	NIDS for Smart cities IoT	2	30	99.90
XGB	Khan	^17^	2022	Elnour et al. HVAC dataset	Attack detection for HVAC	2	24	99.98
XGB	Ashraf	^18^	2022	CICIDS2018, N-BaIoT, KDD Cup 99	NIDS for Blockchain enabled IoT Healthcare	2	10	98.96
XGB/DT	Alissa	^19^	2022	UNSWNB15	Botnet attack detection in IoT	2	40	94.00
XGB LGB	Garg	^20^	2022	BoT-IoT, IoT-23, CICDDoS-19	Attacks Identification: IoT attacks and DDoS attacks	2	35	94.49 94.79
LGB PSO-LGB GSA-LGB	Bhoi	^21^	2022	IoT dataset	Identification of Malicious Access in IoT Network	2	13	99.99 100.0 100.0
HGB	Saied	^22^	2025	N-BaIoT	IoT Botnet Attack Detection	2	115	99.99
HGB	Saied	^23^	2023	N-BaIoT	IoT Botnet Attack Detection	3	115	99.99

## Material and methods

This section introduces the methodology of conducting this study. It proposed a conceptual framework for integrating XAI techniques into the detection of botnet activities in IoT environments. Our framework aims to enhance the interpretability and transparency of the botnet detection system. By integrating XAI, we provide deeper insights into the factors driving the classification characteristics of benign and malicious network traffic in the IoT environment. We describe the dataset used for evaluation, the dataset preprocessing phase, model evaluation phase, the integration of XAI techniques, and the evaluation methodology employed to assess the interpretability and performance of the proposed framework.

### Proposed framework

**Fig. 1:**
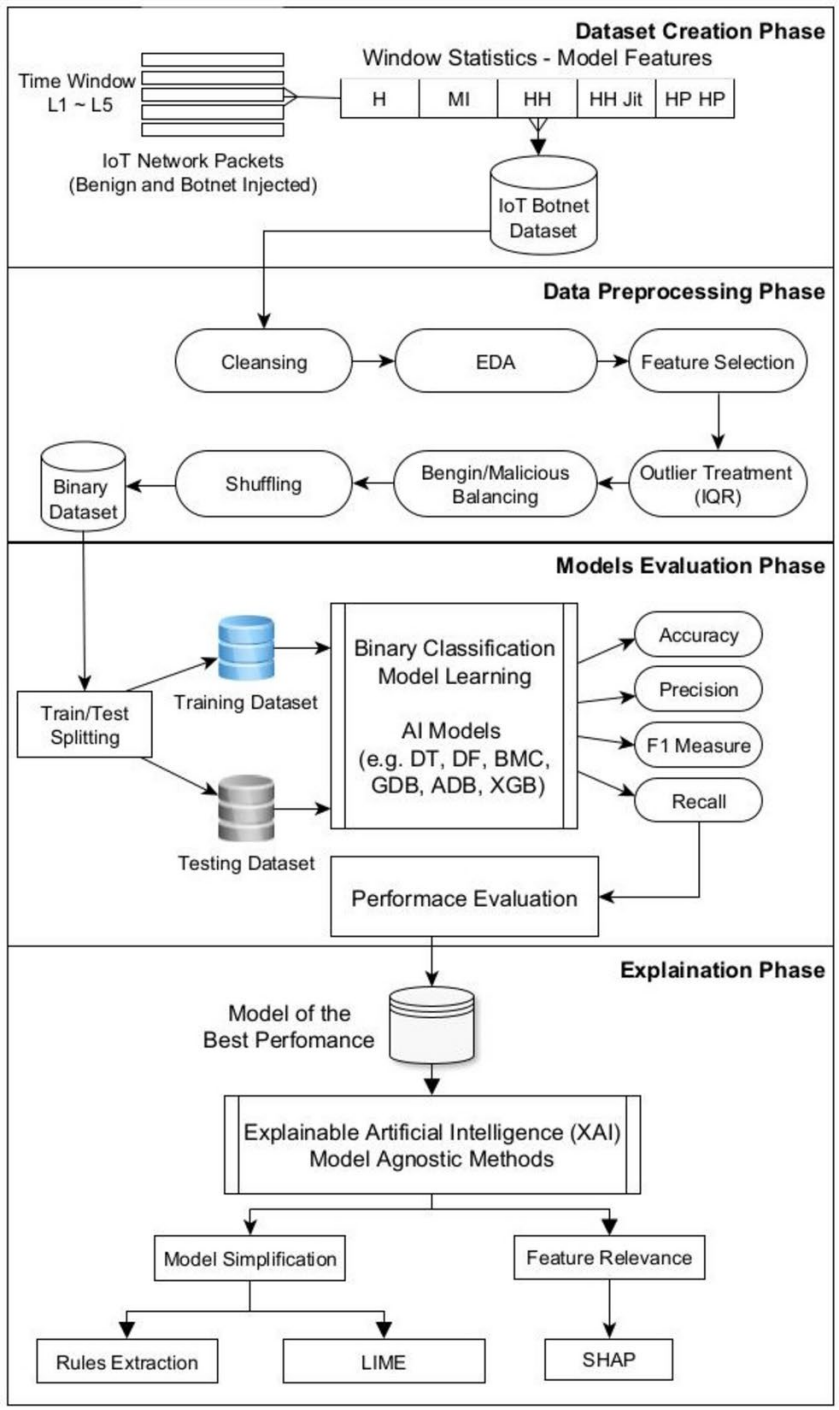
Proposed XAI methodology framework.

Figure 1 illustrates the proposed conceptual framework. It consists of four primary phases. The first phase is the dataset creation. It includes six steps:

1. Sniffing benign IoT network traffic.
2. Injecting botnet malware.
3. Sniffing malicious network traffic.
4. Labelling traffic.
5. Applying traffic statistics.
6. Storing traffic statistics.

The second phase is the dataset preprocessing. It is an essential step in preparing data for model learning. It involves cleaning, transforming, and reformatting the raw data to make it suitable for use in an AI model.

The quality of the preprocessing step plays a crucial role in determining the performance of the machine learning algorithm. This step involves six stages:

1. Dataset cleansing
2. Analyzing the dataset using exploratory data analysis (EDA)
3. Feature selection to select the most relevant features
4. Outliers treatment
5. Dataset balancing
6. Shuffling

The third phase is the models evaluation. It focuses on learning several algorithms and comparative evaluating them. It includes four steps:

1. Dataset splitting into two subsets: a training set and a test set.
2. Models learning: training set is used to train the model.
3. Models testing: test set is used to evaluate the performance of the model on new, unseen data. The test set is utilized to estimate the model’s generalization error, which represents the expected error rate on new data.
4. Models evaluation: evaluating each model performance and forming an evaluation matrix. Based on the evaluation results, the model of the best performance will be selected.

The fourth phase is integrating the selected model with XAI techniques. XAI is applied to the best performing model to add the interpretability in the black-box model. Two XAI explanations types are used: model simplification and feature relevance. Explanation by simplification provides explanation through rule extraction and distillation. Feature relevance explanation provides explanation through ranking or measuring the influence each feature has on a prediction output.

The following is the pseudo code for the proposed framework:

**Algorithm:**
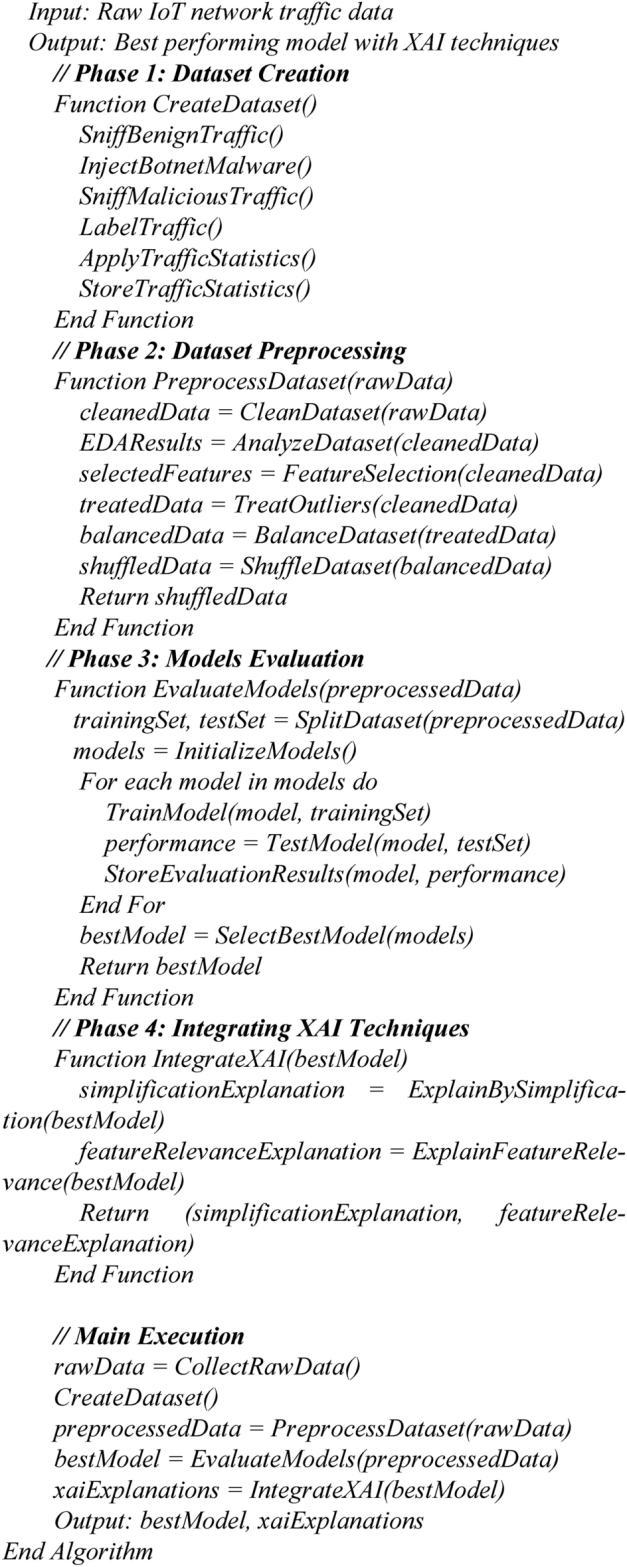
*Proposed framework*

Explanation of the Pseudocode:

*CreateDataset*: This function encapsulates the steps for creating the dataset from both benign and malicious traffic.

*PreprocessDataset*: This function outlines the preprocessing steps, ensuring the data is cleaned and formatted appropriately for model training.

*EvaluateModels*: This function describes how to split the dataset, train different models, and evaluate their performance.

*IntegrateXAI*: This function integrates explainable AI techniques into the best-performing model, providing interpretability.

The Main Execution section brings together all phases, leading to the final output of the best model and its explanations.

### Dataset description

This section describes the dataset used in the experimental framework. N-BaIoT^24^ dataset is selected for training and evaluation purposes as it is a widely accepted as benchmark sequential dataset. It contains realistic network traffic and a variety of attack traffic. It was suggested by Meidan et al.^25^ through gathering traffic of nine commercially available IoT devices authentically infected by Mirai and Bashlite malware. The devices were 2 smart doorbells, 1 smart thermostat, 1 smart baby monitor, 4 security cameras and 1 webcam. The traffic data was captured during normal device execution and after infection with malware. The network sniffing utility was employed to capture the traffic in raw network traffic pcap format, typically achieved through port mirroring. Five features were extracted from the network traffic, as summarized in Table 2. For each of these five features, three or more statistical measures were computed for data aggregation, resulting in a total of 23 features. These 23 distinct features were calculated over five separate time windows (100 ms, 500 ms, 1.5 s, 10 s, and 1 min).

**Table 2:** Dataset attributes information.

Stream aggregation designation	Stream aggregation description	Stream characteristics (statistical aggregation functions)							Count	Time frame	Features
		Weight	Mean	Variance/Standard deviation	Magnitude	Radius	Covariance	Correlation coefficient			
H	Host Source IP	✓	✓	Variance	x	x	x	x	3	5	15
MI	Host Source IP + MAC	✓	✓	Variance	x	x	x	x	3	5	15
HH	Host-to-host channel (Source IP to destination IP)	✓	✓	Std	✓	✓	✓	✓	7	5	35
HH_Jit	Host-to-host channel jitter	✓	✓	Variance	x	x	x	x	3	5	15
HpHp	Host port to Host port channel (IP + Socket)	✓	✓	Std	✓	✓	✓	✓	7	5	35
Tot. Traffic characteristics									23	Tot. Features	115

The use of time windows makes this dataset suitable for stateful Intrusion Detection Systems (IDS), resulting in a total of 115 features. The dataset consists of instances of network traffic data categorized into three groups: normal traffic (Benign data), Bashlite infected traffic, and Mirai infected traffic. Each data instance is represented by 115 features derived from 23 different traffic characteristics across five different time frames. Table 2 provides an abstracted overview of the dataset attributes.

### Dataset preprocessing

The attacks executed by botnets include Scan, which aims to discover vulnerable devices; flooding, which utilizes SYN, ACK, UDP, and TCP flooding techniques; and combo attacks involving opening connections and sending junk data. Figure 3 illustrates the unbalanced nature of the N-BaIoT dataset. Therefore, a subset of the dataset was selected to form a balanced binary class labeled dataset. All instances of benign traffic, totaling 555,932 instances, were included, while the remaining malicious traffic datasets were merged. To achieve a balanced dataset, an equal number of benign instances were selected from the malicious traffic. As a result, the balanced dataset consisted of a total of 1,111,864 instances, as indicated in Table 3.

**Table 3:** Dataset balancing.

Dataset	Classifier	Class	Training set	Testing set
1,111,864	Benign	555,932	889,491	222,372
	Malicious	555,932		

Subsequently, the dataset was randomly shuffled to randomize the order of the training data before feeding it into the learning algorithms. Shuffling is performed to prevent any patterns in the data from influencing the learning algorithm’s order.

### Evaluation metrics

In conducting a thorough performance evaluation, it is crucial to consider various metrics beyond just accuracy. Effective detection requires not only the accurate identification of attacks but also the reduction of false positives. The following four evaluation metrics are utilized:

*Accuracy*: This metric assesses the overall correctness of the classification model and is calculated as the ratio of correctly classified instances to the total number of instances.

$$Accuracy= \frac{TP+TN}{TP+TN+FP+FN}$$

*Precision*: This represents the ratio of true positive predictions to all positive predictions made by the model.

$$Precision= \frac{TP}{TP+FP}$$

*Recall*: This measures the model’s ability to correctly identify positive instances from all actual positive cases.

$$Recall (Sensitivity)= \frac{TP}{TP+FN}$$

*F1 Score*: This combines precision and recall into a single metric, providing a balanced assessment of the model’s performance.

$$F1 Score= \frac{2 \times Precision \times Recall}{Precision+Recall}$$

All these metrics range from 0 to 1, with higher values indicating superior classification performance. Additionally, two further performance metrics training time and testing time are included for a comprehensive comparative evaluation.

### Models evaluation

The present study builds upon our prior research^2^, which focused on the empirical evaluation of six tree-based algorithms: DT, RF, BMC, ADB, GDB, and XGB. The results of previous study^2^ is shown in Table 4. It shows that RF model outperforms the other models in all evaluation measures.

**Table 4:** Evaluation results.

Classifier	Accuracy	Class	Precision	Recall	F1 score
DT	0.999941	Benign	0.999946	0.999937	0.999941
		Malicious	0.999936	0.999945	0.999941
BMC	0.999964	Benign	0.999946	0.999982	0.999945
		Malicious	0.999981	0.999945	0.999963
RF	**0.999982**	Benign	**0.999964**	**1.000000**	**0.999982**
		Malicious	**1.000000**	**0.999963**	**0.999981**
ADB	0.999968	Benign	0.999946	0.999991	0.999969
		Malicious	0.999990	0.999945	0.999968
GDB	0.999892	Benign	0.999784	1.000000	0.999892
		Malicious	1.000000	0.999783	0.999891
XGB	0.999914	Benign	0.999883	0.999946	0.999914
		Malicious	0.999945	0.999882	0.999914

Significant values are in bold

Table 5 compares the performance of this study with a previous study^22^. The previous study has been selected as it utilized the same dataset and same number of classes. The results show that the RF algorithm outperforms the adopted algorithm HGB in the previous study in all the evaluation metrics.

**Table 5:** Results comparison.

Study	Dataset	No of classes	Classifier	Class	Accuracy	Precision	Recall	F1 score
^22^	N-BaIoT	2	HGB	Benign	0.999977	0.999964	0.999991	0.999977
				Malicious		0.999990	0.999963	0.999977
This study	N-BaIoT	2	RF	Benign	0.999982	0.999964	1.000000	0.999982
				Malicious		1.000000	0.999963	0.999981

### Integration of XAI techniques

XAI refers to the techniques and methods used to make ML models more transparent and interpretable. The goal of XAI is to provide explanations for the decisions made by AI models, enabling users to understand and trust the reasoning behind those decisions.

To enhance the interpretability and transparency of the best performing model (Random Forest model) in the context of botnet detection, we apply XAI techniques. Explainable AI includes a collection of techniques and approaches that seek to offer transparent and comprehensible explanations for the decisions made by AI and ML models, ensuring their interpretability to humans. Specifically, we utilized two types of XAI explanations: model simplification and feature relevance.

#### Model simplification explanation

It provides insights into the decision-making process of enigmatic or black-box model by extracting and distilling rules. This explanation type enables us to obtain a simplified representation of the complex model’s behavior.

To generate simplification explanations for individual predictions made by the RF model, we employed two techniques: rule extraction and distillation technique, and local interpretable model-agnostic explanations (LIME) technique.

By leveraging model simplification explanations, we can gain insights into the key factors and conditions that contribute to the model’s predictions for botnet detection in the IoT environment. This enables security analysts and domain experts to better understand the decision-making process and identify potential vulnerabilities or biases within the model.

Rule extraction and distillation techniques aim to identify a set of rules that mimic the black-box model’s behavior while maintaining a high level of interpretability. The extracted rules provide a human-understandable representation of the decision logic employed by the model.

LIME is first introduced by Ribeiro, Marco Tulio Guestrin, Carlos^26^. LIME operates by approximating the decision boundaries around specific instances and identifying the most influential features contributing to the model’s predictions. By generating explanations in the form of feature importance weights, LIME provides insights into the factors driving the classification outcomes and highlights the distinguishing characteristics of benign and malicious network traffic in the IoT environment. LIME creates an interpretable local model around the instance of interest, which can be used to understand the model’s decision. It aims to explain the predictions of black-box models by approximating them with interpretable models, thus providing insights into the decision-making process.

#### Feature relevance explanation

It provides an understanding of the influence that each feature has on the model’s prediction outputs. This type of explanation ranks or measures the relevance of individual features in driving the model’s decision-making process.

To generate feature relevance explanations, we employed SHapley Additive exPlanations technique (SHAP). SHAP is first introduced by Lundberg and Lee^27^. These techniques utilize game theory principles through assigning importance scores to each feature based on their impact on the model’s predictions. The higher the importance score, the more influential the corresponding feature is in determining the output.

SHAP is based on cooperative game theory that provides a unified approach to explain the output of any machine learning model. It assigns each feature in the input a "SHAP value," which represents the contribution of that feature to the model’s prediction for a specific instance. SHAP values provide a global perspective on feature importance and also enable instance-level explanations.

By utilizing feature relevance explanations, we can identify the most critical features for detecting IoT botnet activities. This information aids in understanding the underlying characteristics and patterns associated with botnet behavior in the IoT environment. It allows the intrusion detection process to be more transparent and improves interpretability of the detection system.

## Experiments and results

In this section, we present the experiments conducted to evaluate the effectiveness and interpretability of the proposed framework for IoT botnet detection, incorporating XAI techniques.

### Experimental setup

We implemented the proposed framework using Python environment (version 3.9.7) and leveraged the Scikit-learn library for building the classification models. The RF algorithm was selected as the best performing model based on preliminary evaluations. To assess the interpretability of the framework, we applied the selected XAI techniques, including model simplification and feature relevance explanations, to the trained RF model. In our experimental study focusing on botnet detection in IoT systems, we employed rule extraction, Shapley, and LIME techniques to gain insights into the prediction process and enhance interpretability. Integrating the XAI techniques was implemented using LIME (version 0.2.0.1) for local explanation and SHAP (version 0.41.0) for global explanation.

Rule extraction techniques are employed to extract human-readable rules from the machine learning model. These rules served as logical representations of the decision boundaries and conditions for botnet detection. The extracted rules not only facilitated interpretability but also provided a transparent and actionable framework for identifying botnet activity in IoT systems.

LIME is applied to explain the predictions made by our machine learning model on individual instances. The LIME explanations provided valuable information regarding the contribution and importance of different features in determining the botnet detection outcome. This enabled us to understand the decision-making process at a local level and identify key indicators of botnet activity in IoT devices.

Utilized Shapley values measure the impact of each feature on the prediction across multiple instances. By calculating the Shapley values, we were able to quantify the individual relevance of features and identify those with the most significant influence on the botnet detection outcome. This global interpretability measure complemented the local explanations obtained through LIME, providing a more comprehensive understanding of the model’s behavior.

### Results and interpretations

Through the integration of XAI techniques, we gained valuable insights into the model’s decision-making process. The model simplification explanations revealed a set of interpretable rules that captured the key conditions and features contributing to the classification outcomes. These rules facilitated the identification of specific patterns associated with botnet activities. In the context of botnet detection in IoT, rule extraction provides interpretable and actionable guidelines for identifying botnet activity. The extracted rules represent logical conditions and decision boundaries that can be easily understood and implemented by security practitioners. These rules may include specific combinations of features or thresholds that indicate the presence of botnet behavior. By leveraging the extracted rules, security professionals can proactively detect and respond to botnet attacks in IoT systems, enhancing the overall security posture. The rules are extracted from the RF model using the global surrogate model and the top 8 extracted rules are mentioned in Table 6.

**Table 6:** Top 8 extracted rules.

Rule ID	Condition	Outcome	Probability (%)	Samples
1	If (HpHp_L0.01_weight > 1.981) & (H_L3_weight < = 68.244) & (HH_jit_L1_variance < = 956468789248.0)	Benign	99.78	253,474
2	If (HpHp_L0.01_weight < = 1.981) & (HH_L0.01_radius < = 147.878) & (MI_dir_L0.01_mean < = 74.806)	Malicious	99.70	175,228
3	If (HpHp_L0.01_weight < = 1.981) & (HH_L0.01_radius < = 147.878) & (MI_dir_L0.01_mean > 74.806)	Malicious	90.78	94,127
4	If (HpHp_L0.01_weight > 1.981) & (H_L3_weight < = 68.244) & (HpHp_L5_radius < = 2.102)	Malicious	99.96	15,651
5	If (HpHp_L0.01_weight < = 1.981) & (HH_L0.01_radius > 147.878) & (HH_L0.1_mean < = 530.309)	Benign	99.80	15,553
6	If (HpHp_L0.01_weight > 1.981) & (H_L3_weight < = 68.244) & (HH_jit_L1_variance > 956,468,789,248.0)	Malicious	55.88	4,597
7	If (HpHp_L0.01_weight < = 1.981) & (HH_L0.01_radius < = 147.878) & (HH_L0.1_mean > 530.309)	Malicious	99.45	2,619
8	If (HpHp_L0.01_weight > 1.981) & (H_L3_weight > 68.244) & (HpHp_L5_radius > 2.102)	Benign	98.79	1,363

The feature relevance explanations highlighted the most influential features in detecting IoT botnet behaviors. By ranking the features based on their relevance scores, we identified the key characteristics that differentiate benign and malicious network traffic. This information can be used to prioritize and focus on specific features during further investigations and feature engineering efforts. Shapley values provide a global interpretability measure by quantifying the contribution of each feature across multiple instances. In the context of botnet detection in IoT, Shapley values help us understand the relative importance of different features in determining the overall botnet detection outcome. By calculating the Shapley values, we can identify the features that have the most significant impact on the detection process. This insight enables us to prioritize and allocate resources for monitoring and mitigating the most influential features, thereby enhancing the effectiveness of botnet detection in IoT systems.

**Fig. 2:**
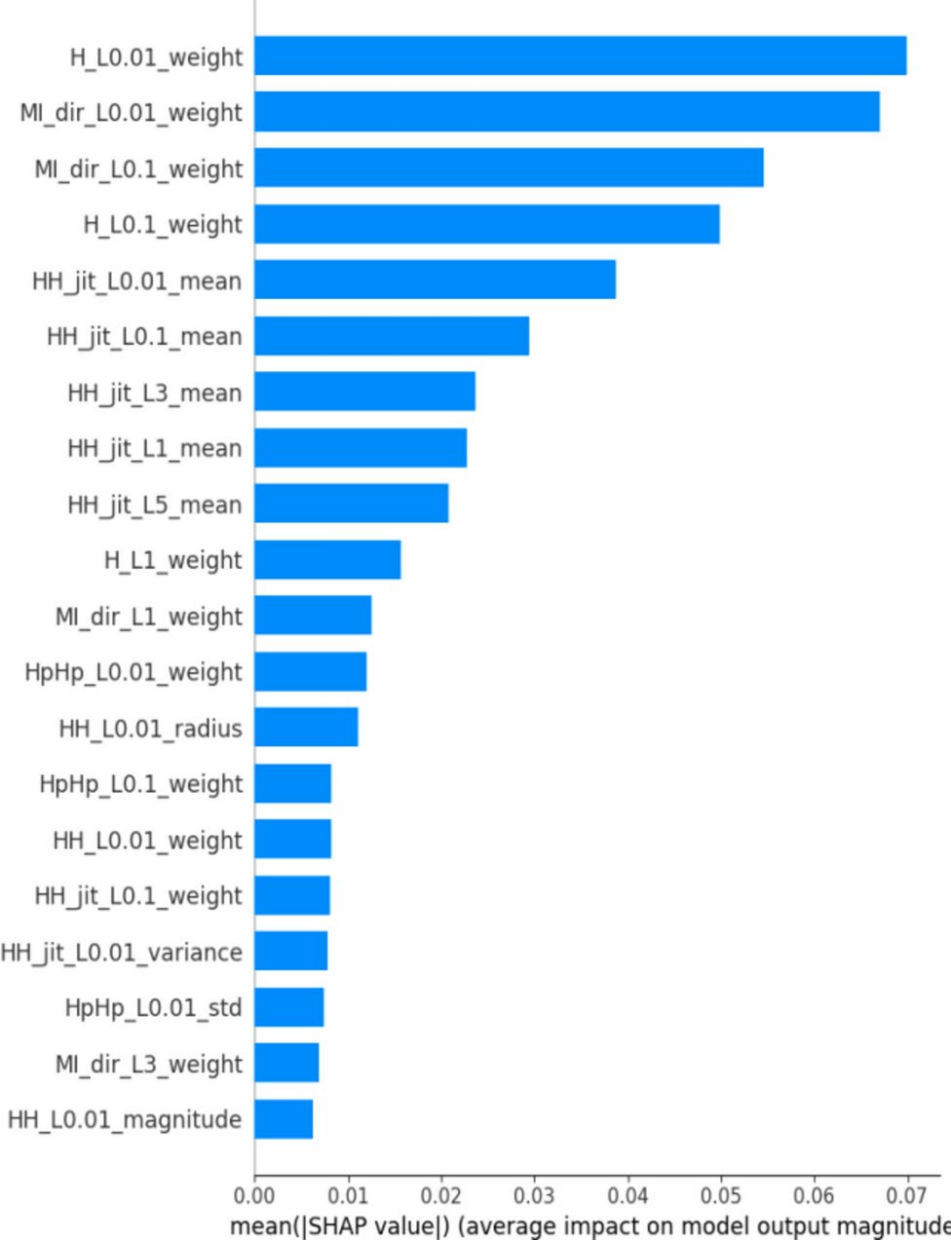
Global explanation of random forest classifier using SHAP.

To calculate the Shapley value, SHAP considers all possible permutations of features and evaluates their contributions. For each permutation, it systematically determines the marginal contribution of each feature by comparing the model’s predictions when the feature is included or excluded. One of the fundamental properties of Shapley values is that SHAP values of all the input features will always sum up to the difference between baseline model output and the current model output for the prediction being explained. The easiest way to see this is through a waterfall as shown in Fig. 2.

**Fig. 3:**
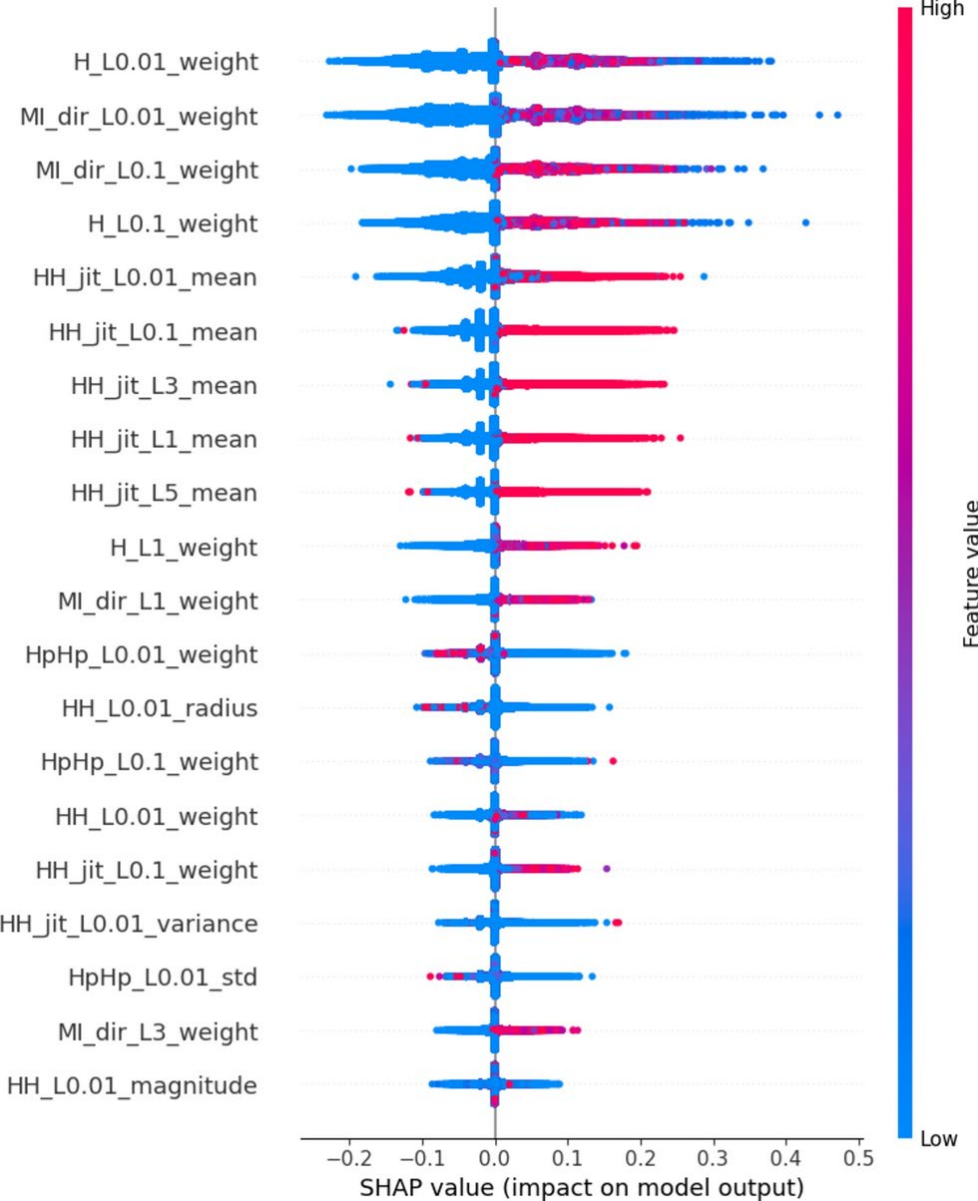
Bee swarm chart of random forest classifier using SHAP.

In this plot, features are ranked by their mean SHAP values all the instances (rows) of the dataset showing the most important features at the top and the least important ones at the bottom. This helps to understand the impact of each feature on the model’s predictions. To provide a more granular overview of the impact of each feature on a specific predicted class, Fig. 3 shows a bee swarm chart to summarize the entire distribution of SHAP values for the most 20 important features. Y-axis represents the features ranked by their average absolute SHAP values. X-axis represents SHAP values. Positive values for a given feature push the model’s prediction closer to the label being examined (label = 1 or malicious traffic). In contrast, negative values push towards the opposite class (label = 0 or benign traffic). The color represents the value of the feature from low to high. Overlapping points are jittered in the y-axis direction, so we get a sense of the distribution of the Shapley values per feature.

By examining the Shapley chart, we can identify the features with the highest positive or negative Shapley values. Features with high positive values indicate a positive influence on the botnet detection outcome, meaning their presence or certain values contribute to identifying botnet activity. On the other hand, features with high negative values suggest a negative influence, indicating that their absence or specific values are indicative of botnet presence. By understanding the impact of each feature through the Shapley chart, we can prioritize monitoring and mitigation efforts accordingly, focusing on the most influential features for effective botnet detection in IoT systems.

We can observe that the red and blue colors occupy half of the horizontal bee swarm for each class. This means that each feature has an almost equal impact on the classification of both malicious (label = 1) and benign (label = 0) classes. However, H_L0.01_weight, MI_dir_L0.01_weight and MI_dir_L0.1_weight are the first three features with the most predictive power. On the other hand, HpHp_L0.01_std, MI_dir_L3_weight, and HH_L0.01_magnitude do not contribute as much as the first three features.

Figure 3 shows that host-to-host jitter feature is clearly able to discriminate the malicious and benign traffic. It shows that the network traffic with a high mean host-to-host jitter HH_jit_L0.01_mean, HH_jit_L0.1_mean, HH_jit_L3_mean, HH_jit_L1_mean, HH_jit_L5_mean (red dots) level is likely to be classified as malicious traffic (positive alarm), while a low level leads to not being classified as malicious.

Similarly, the network traffic of low level host source IP weight (H_L0.01_weight) is more likely to be classified as benign traffic. However, the model seems uncertain about classifying the high level of some features e.g. H_L0.01_weight, MI_dir_L0.01_weight, MI_dir_L0.1_weight, and H_L0.01_weight.

The summary plot gives first indications of the relationship between features and their impact on the model predictions. But to deal with this ambiguity for those features, one way is though using the dependence plot to gain more insights. In contrast to summary plots, dependence plots depict the correlation between a particular feature and the predicted outcome for individual instances in the dataset. The purpose of this analysis extends beyond obtaining detailed insights and verifying the significance of the feature under examination.

It involves confirming or questioning the conclusions drawn from summary plots or other global measures of feature importance. It can discover the relationship between the model prediction and a certain feature whether it is linear, monotonic, or more complex.

**Fig. 4:**
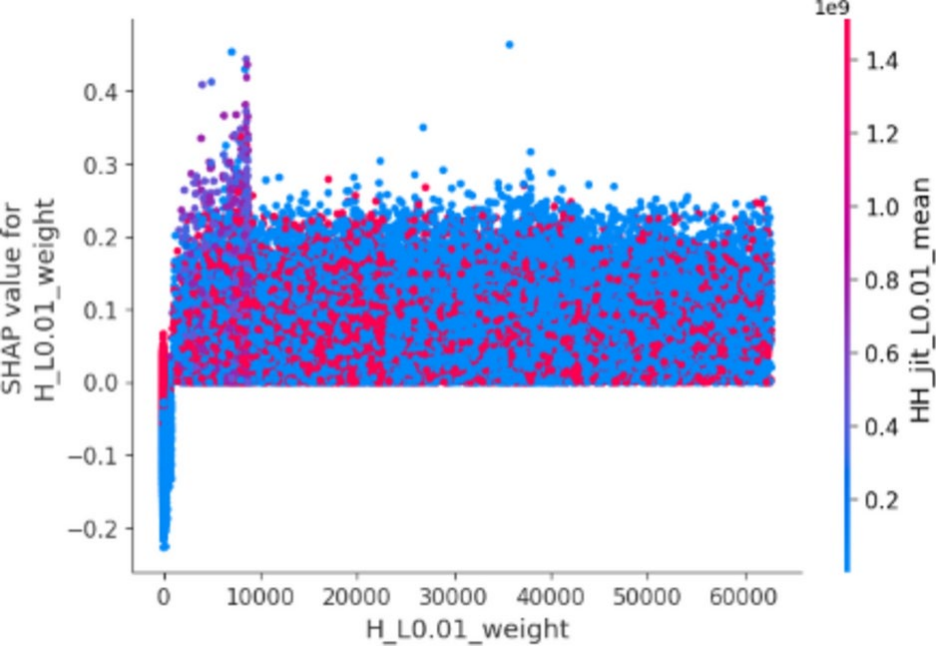
Dependence chart for H_L0.01_weight.

**Fig. 5:**
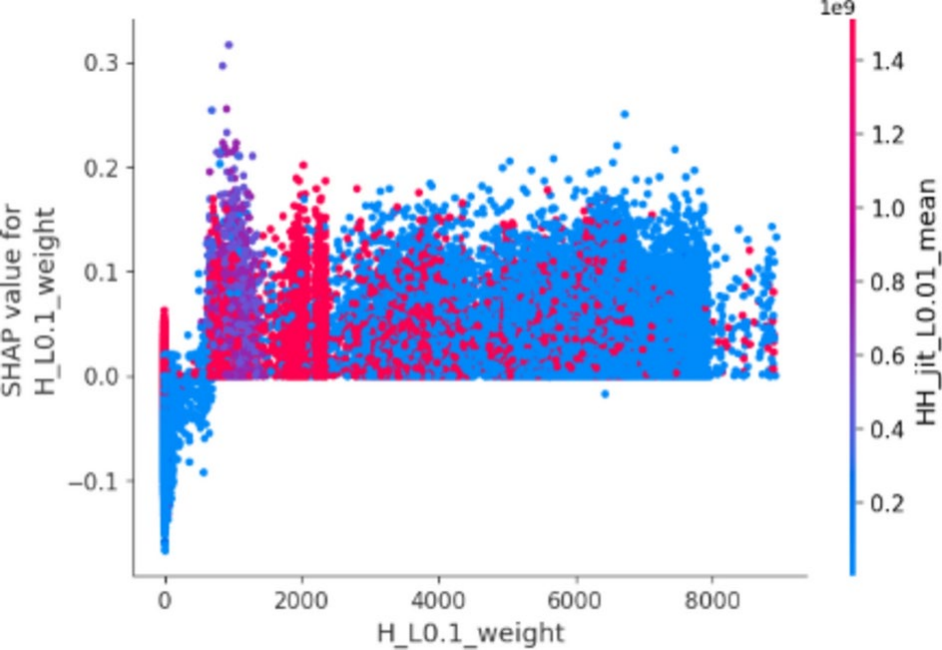
Dependence chart for H_L0.1_weight.

**Fig. 6:**
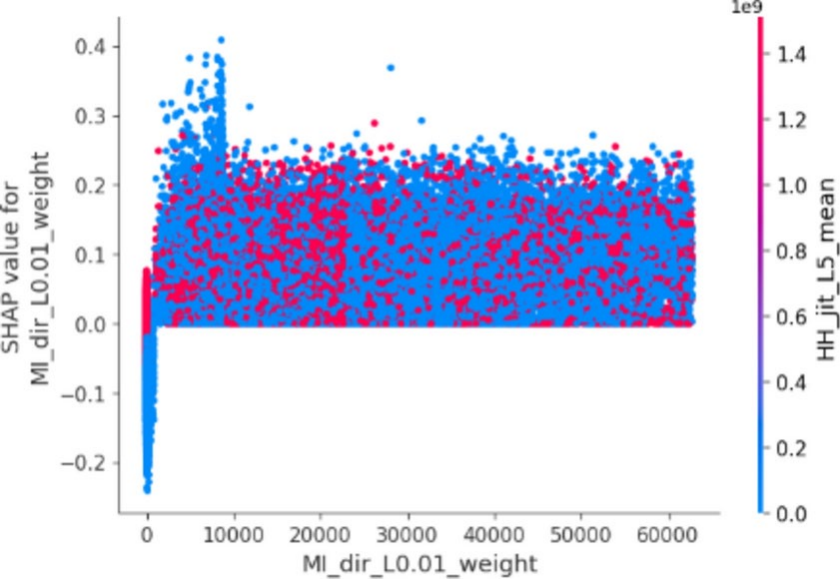
Dependence chart for MI_dir_L0.01_weight.

**Fig. 7:**
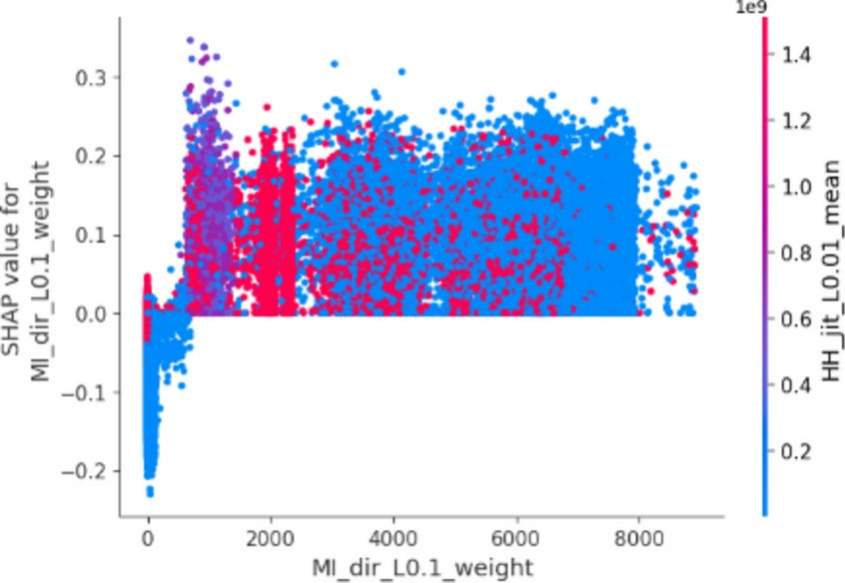
Dependence chart for MI_dir_L0.1_weight.

Figures 4, 5, 6 and 7 show the dependence plots for the H_L0.01_weight, H_L0.1_weight, MI_dir_L0.01_weight, and MI_dir_L0.1_weight, respectively. The figures plot the feature values on the x-axis and the corresponding Shapley values on the y-axis. Each dot is a single prediction (row) from the dataset.

LIME was employed to provide deeper interpretability for the model and gain deeper insights into the key indicators and factors that contribute to the detection of botnets in the IoT ecosystem.

The chart of LIME explanations provides insights into the local decision-making process for individual instances in the botnet detection task. Each instance is represented on the chart, along with the contribution of different features towards the prediction. By analyzing the LIME explanations, we can identify the specific features that contribute most significantly to the prediction of botnet activity for each instance. This information helps in understanding the unique characteristics and indicators of botnet behavior in IoT devices.

The LIME chart consists of three main components that provide insights into the explanation of individual predictions in the context of botnet detection in IoT:

Original instance:

The LIME chart starts by displaying the original instance or data point for which the prediction is being explained. This refers to a specific IoT device or data sample that is being analyzed for botnet detection. The original instance represents the input features and their corresponding values that are fed into the machine learning model for prediction.

Feature contributions:

The LIME chart includes the feature contributions, which represent the contribution of each feature in determining the botnet detection outcome for the original instance. Each feature is assigned a corresponding bar or line on the chart, with its length or height indicating the magnitude or strength of its contribution. Positive contributions suggest that the feature value is indicative of botnet activity, while negative contributions imply a feature value that is indicative of the absence of botnet behavior.

Overall explanation:

The LIME chart provides an overall explanation of the prediction for the original instance. This explanation summarizes the combined effect of all the feature contributions and provides a holistic understanding of why the prediction was made. It helps in interpreting the decision process of the machine learning model and provides insights into the factors or features that contribute to identifying botnet activity.

By examining the original instance, feature contributions, and overall explanation in the LIME chart, we can gain a better understanding of how specific features influence the botnet detection outcome for individual instances. This information is crucial for interpreting and validating the predictions made by the machine learning model. It allows us to identify the key indicators or patterns associated with botnet behavior in the IoT context and aids in building trust, transparency, and interpretability in the botnet detection process.

**Fig. 8:**
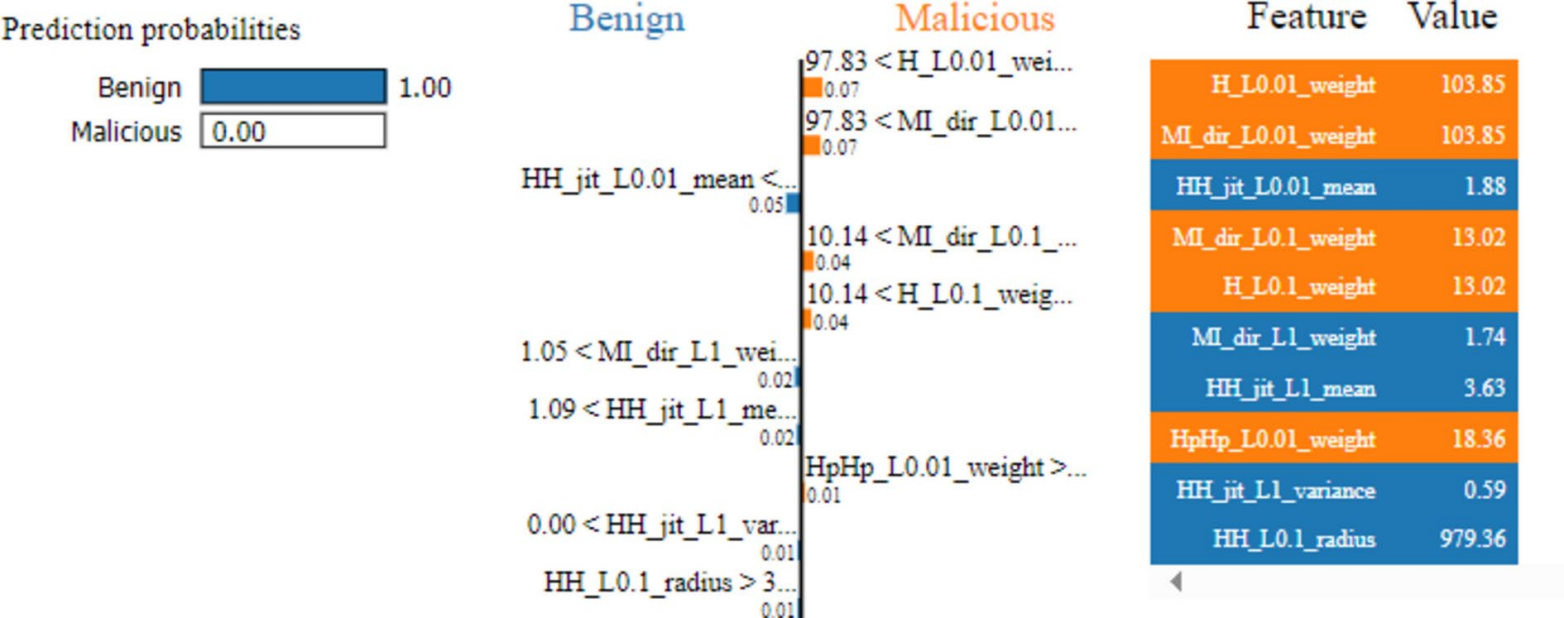
LIME chart for a test vector of benign traffic.

Two cases are used to demonstrate how LIME model can interpret the individual predictions. Figure 8 explains the first case of benign classified traffic. It interprets the decisions behind the prediction for the first test vector by displaying the top 10 features which contributed towards the said model’s prediction. The colors blue and orange are depicting negative (benign traffic) and positive (malicious traffic), respectively. This denotes the prediction given by our prediction RF model for the given test vector that has been classified as benign traffic. Five features out of the ten contributed in predicting that the traffic is benign.

**Fig. 9:**
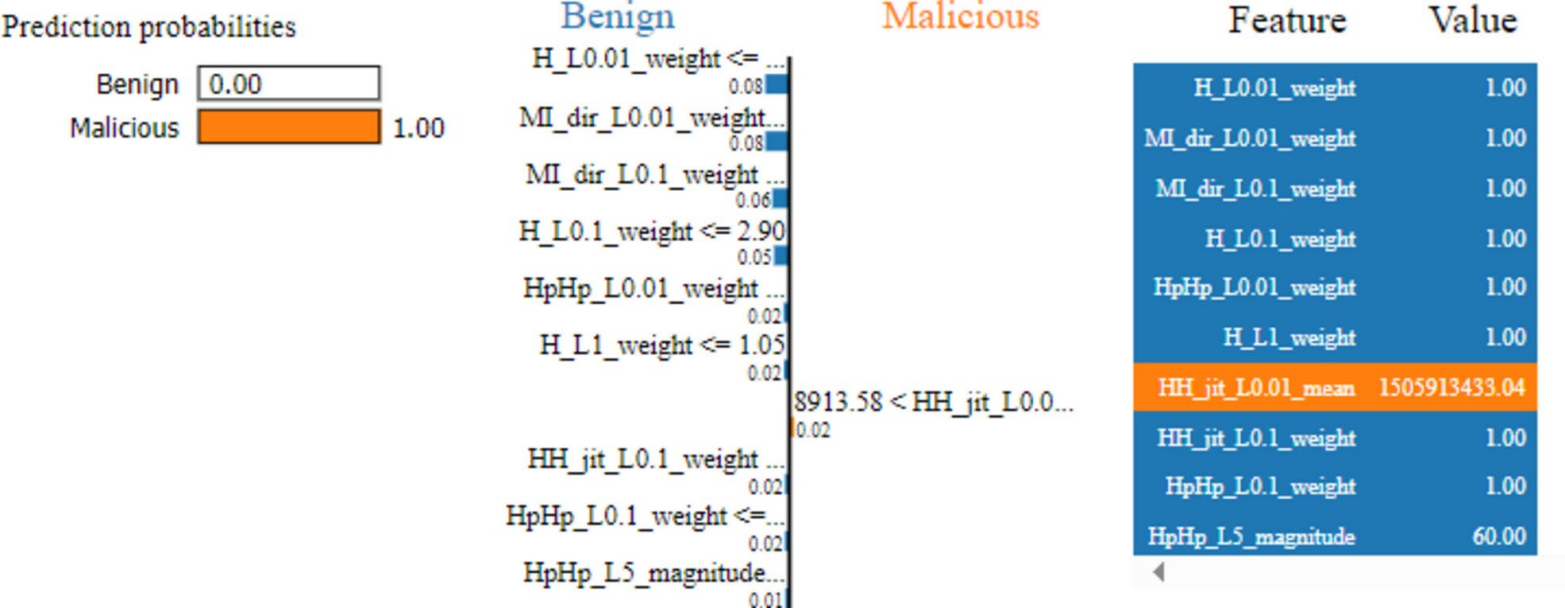
LIME chart for a test vector of malicious traffic.

Figure 9 explains the second case of malicious classifies traffic. It shows that the main reason led the model to classify the traffic as malicious is due to the value of the host-to-host jitter feature (HH_jit_L01.01_mean). However, the rest 9 features of the most 10 important features indicate that the traffic is benign; the most significant feature for the model is the host-to-host jitter mean feature.

The LIME chart can reveal patterns, correlations, or anomalies in feature contributions across instances, which may provide additional insights into the underlying mechanisms of botnet detection. It assists in identifying common features associated with botnet presence and understanding the variations within the IoT ecosystem.

## Conclusion and future work

The paper presented an experimental evaluation for adopting XAI techniques in botnet intrusion detection in IoT through utilizing a well-known standard dataset N-BaIoT. The combination of LIME, Shapley values, and rule extraction techniques proved to be highly effective in enhancing interpretability and understanding the botnet detection process in IoT systems. LIME provides local interpretability, allowing us to analyze individual instances, while Shapley values offer a global perspective on feature importance. Rule extraction techniques provide actionable rules that guide the detection process. Together, these techniques provide deep insights into the underlying mechanisms of botnet detection in IoT systems and enabling effective security measures. The insights gained from these techniques can aid in fine-tuning the model, improving security measures, and enabling proactive mitigation strategies against botnet attacks in IoT environments.

While XAI techniques enhance the interpretability of AI models, they also introduce vulnerabilities that adversaries can exploit. It is crucial to critically assess the potential risks associated with revealing important features and to develop strategies that enhance model security without sacrificing the interpretability that is essential for trust and accountability in AI systems. Future work should aim to strike a balance between interpretability and security, ensuring that AI systems remain both transparent and robust in the face of evolving adversarial tactics, particularly in critical applications such as botnet detection in IoT environments.

## Data Availability

The dataset of Median et al. that support the findings of this study is available in Kaggle repository with the identifier [doi: “https://doi.org/10.1109/MPRV.2018.03367731”] [“https://www.kaggle.com/datasets/mkashifn/nbaiot-dataset/discussion/176689”].

## References

[1] Saied, M., Guirguis, S. & Madbouly, M. Review of artificial intelligence for enhancing intrusion detection in the internet of things. In *Engineering Applications of Artificial Intelligence*, vol. 127, 107231. (Elsevier Ltd, 2024). 10.1016/j.engappai.2023.107231

[2] Saied, M. & Guirguis, S. Evaluation of tree based machine learning algorithms for network intrusion detection in IoT. In *IEEE IT Prof.* (2023).

[3] Angrishi, K. Turning internet of things (IoT) into internet of vulnerabilities (IoV) : IoT botnets 1–17. arXiv Prepr. (2017). Available: http://arxiv.org/abs/1702.03681

[4] Longo, L. et al. Explainable artificial intelligence: Concepts, applications, research challenges and visions to cite this version: HAL Id: hal-03414756 explainable artificial intelligence: Concepts, applications, research challenges and visions. In *4th International Cross- Domain Conference for Machine Learning and Knowledge Extraction (CD-MAKE), Aug 2020, Dublin, Ireland *0–16 (2021).

[5] Gerlings, J., Shollo, A. & Constantiou, I. Reviewing the need for explainable artificial intelligence (xAI). In *Proceedings of the 54th Hawaii International Conference on System Sciences|2021 Reviewing* 1284–1293 (2021).

[6] Doshi, R., Apthorpe, N. & Feamster, N. Machine learning DDoS detection for consumer internet of things devices. In *Deep Learning and Security Workshop (DLS). *(IEEE, 2017).

[7] Anthi, E., Williams, L., Słowi, M., Theodorakopoulos, G. & Burnap, P. A supervised intrusion detection system for smart home IoT devices. *IEEE Internet Things J.* **4662**, 1–13. 10.1109/JIOT.2019.2926365 (2019).

[8] Goyal, M., Sahoo, I & Geethakumari, G. HTTP botnet detection in IOT Devices using network traffic analysis. In *2019 International Conference on Recent Advances in Energy-Efficient Computing and Communication (ICRAECC)* 1–6 (2019).

[9] Chaudhary, P. & Gupta, B. B. DDoS detection framework in resource constrained internet of things domain. In *2019 IEEE 8th Global Conference on Consumer GCCE* 675–678 (2019). 10.1109/GCCE46687.2019.9015465

[10] Alrashdi, I., Alqazzaz, A., Aloufi, E., Alharthi, R., Zohdy, M. & Ming, H. AD-IoT: Anomaly detection of IoT cyberattacks in smart city using machine learning. In *2019 IEEE 9th Annual Computing and Communication Workshop and Conference* 305–310 (2019). 10.1109/CCWC.2019.8666450

[11] Moustafa, N. & Slay, J. UNSW-NB15: A comprehensive data set for network intrusion detection systems (UNSW-NB15 network data set). In *2015 Military Communications and Information Systems Conference (MilCIS)* 1–6 (2015). 10.1109/MilCIS.2015.7348942

[12] Thamilarasu, G., Odesile, A. & Hoang, A. An intrusion detection system for internet of medical things. *IEEE Access.*10.1109/ACCESS.2020.3026260 (2020).

[13] Hammoudeh, M. & Aljaberi, S. M. Modeling of deep learning based intrusion detection system in internet of things environment. *J. Cybersecur. Inf. Manag.* **8**(1), 17–25. 10.5281/zenodo.5501286 (2021).

[14] Al Tobi, A. M. & Duncan, I. KDD 1999 generation faults: A review and analysis. *J. Cyber Secur. Technol.* **2**, 1–37. 10.1080/23742917.2018.1518061 (2018).

[15] Kumar, A., Kumar, N. & Shukla, S. K. PeerClear: Peer-to-peer bot-net detection. In *International Symposium on Cyber Security Cryptography and Machine Learning* 279–295 (2019). 10.1007/978-3-030-20951-3_24

[16] Hazman, C., Guezzaz, A., Benkirane, S. & Azrour, M. lIDS-SIoEL: Intrusion detection framework for IoT-based smart environments security using ensemble learning. *Clust. Comput.* **2**, 1–15. 10.1007/s10586-022-03810-0 (2022).

[17] Khan, I. U. et al. A proactive attack detection for heating, ventilation, and air conditioning (HVAC) system using explainable extreme gradient boosting model (XGBoost). *Sensors* **22**(23), 9235 (2022). 36501938 10.3390/s22239235 PMC9740645

[18] Ashraf, E., Areed, N. F. F., Salem, H., Abdelhay, E. H. & Farouk, A. FIDChain: Federated intrusion detection system for blockchain-enabled IoT healthcare applications. *Healthcare* **10**, 6. 10.3390/healthcare10061110 (2022). 10.3390/healthcare10061110 PMC9222634 35742161

[19] Alissa, K. et al. Botnet attack detection in IoT using machine learning. *Comput. Intell. Neurosci.* **2022**, 4515642 (2022). 36238679 10.1155/2022/4515642 PMC9553419

[20] Garg, S., Kumar, V. & Payyavula, S. R. Identification of internet of things (IoT) attacks using gradient boosting: A cross dataset approach. *Telematique* **21**(1), 6982–7012 (2022).

[21] Naik, G. B. B, B., Oram, E. & Vimal, S. Gravitational search optimized light gradient boosting machine for identification of malicious access in IoT network. In *International Conference on Computational Intelligence in Pattern Recognition* 570–579 (2022).

[22] Saied, M., Guirguis, S. & Madbouly, M. IoT cybersecurity: On the use of boosting based approaches for botnet detection. *IEEE IT Prof.* (2024).

[23] Saied, M., Guirguis, S. & Madbouly, M. A comparative study of using boosting-based machine learning algorithms for IoT network intrusion detection. *Int. J. Comput. Intell. Syst.* **16**, 1. 10.1007/s44196-023-00355-x (2023).

[24] Naveed, K., Wu, H. & Abusaq, A. Dytokinesis: A cytokinesis-inspired anomaly detection technique for IoT devices. In *IEEE 45th Conference on Local Computer Networks* 373–376 (2020).

[25] Meidan, Y. et al. N-BaIoT-network-based detection of IoT botnet attacks using deep autoencoders. *IEEE Pervasive Comput.* **17**(3), 12–22. 10.1109/MPRV.2018.03367731 (2018).

[26] Ribeiro, M. T. & Guestrin, C. Why should I trust you ?” Explaining the predictions of any classifier. In *KDD ’16: Proceedings of the 22nd ACM SIGKDD International Conference on Knowledge Discovery and Data Mining* 1135–1144 (2016). 10.1145/2939672.2939778

[27] Lundberg, S. M. & Lee, S. A unified approach to interpreting model predictions. In *31st Conference on Neural Information Processing Systems (NIPS 2017), Long Beach, CA, USA*, 1–10 (2017).

